# Systematic Review of Olaparib in the Treatment of Recurrent Platinum Sensitive Ovarian Cancer

**DOI:** 10.3389/fonc.2022.858826

**Published:** 2022-03-01

**Authors:** Qian Chen, Xiaoli Li, Zhen Zhang, Tong Wu

**Affiliations:** Department of Pharmacy, Beijing Gaobo Boren Hospital, Beijing, China

**Keywords:** olaparib, ovarian cancer, effectiveness, safety, total survival time, adverse reactions

## Abstract

**Objective:**

To systematically evaluate the efficacy and safety of olaparib in the treatment of recurrent platinum-sensitive ovarian cancer.

**Methods:**

The Cochrane Library, PubMed, Chinese Biomedical Literature Database, CNKI, VIP Database, Wanfang Science and Technology Database were searched for randomized controlled trials (RCTs) of olaparib in the treatment of recurrent platinum-sensitive ovarian cancer from the establishment of each database to January 2022. Two reviewers independently evaluated the quality of the literature, extracted the data, and cross-checked the methodological quality. Meta-analysis was performed using RevMan 5.4 software.

**Results:**

A total of 7 RCTs were included, including 2406 patients, There were 1497 patients in treatment groups and 909 patients in the control group. Meta-analysis results showed that in terms of effectiveness, the overall survival time of patients in the olaparib group [HR=1.24, 95%CI(1.06, 1.45), P=0.006]; in terms of safety, for all grades of adverse events (including nausea, fatigue, vomiting, diarrhea, abdominal pain, and headache), [HR=1.54, 95%CI(1.38, 1.71), P=0.0002], for grade 3 or higher adverse events (including nausea, fatigue, vomiting, diarrhea, abdominal pain, and headache), [HR=2.13, 95%CI(1.61, 2.81), P=0.003], there were significant differences compared with the control group, suggesting that the risk of adverse reactions in the experimental group was higher than that in the control group. Subgroup analysis showed that only abdominal pain, headache and vomiting were not statistically significant, and other adverse reactions were statistically significant.

**Conclusion:**

Based on the existing clinical evidence, olaparib in the treatment of recurrent platinum-sensitive ovarian cancer has a longer overall survival than the control group. It is an ideal regimen, but the incidence of adverse reactions is high.

## Introduction

Ovarian cancer is a common female malignant tumor with high morbidity and mortality, ranking third among gynecological malignant tumors. Approximately 70% of patients relapse within 3 years after completion of first-line chemotherapy, and the average 5-year survival rate of ovarian cancer is lower than that of other tumor types (1, 2). Although patients with ovarian cancer are significantly sensitive to platinum-based chemotherapy agents, many patients experience recurrence and disease progression within 3 years of treatment, highlighting the need for therapies that improve clinical outcomes. In about half of patients with serous ovarian cancer, the homologous recombination repair function of cancer cells is missing, resulting in double stranded DNA damage that cannot be repaired. Homologous recombination repair defects are usually caused by mutations in cancer suppressor genes BRCA1 and BRCA2, which inhibit polyadenyldiphosphate ribose polymerase with single strand DNA breaks mainly through base excision repair pathway, resulting in double strand breaks (3). Olaparib is a poly ADP ribose polymerase (PARP) inhibitor. It shows significant clinical activity in ovarian cancer, especially BRCA1 and BRCA2 mutant cancer. It can inhibit DNA single strand repair, lead to cancer cell apoptosis and improve the effect of chemotherapy. Olaparib has been approved as a maintenance treatment for platinum sensitive patients with recurrent ovarian cancer in the United States and Europe. As the first targeted drug for ovarian cancer, it was approved by China food and drug administration to be listed in China in September 2018 (4). Clinically, it is often used alone or combined with paclitaxel and other conventional drugs to treat ovarian cancer, which has a significant effect and improves the overall survival and clinical benefit rate of cancer patients. However, there is a lack of systematic evaluation of the efficacy and safety of the drug in the treatment of ovarian cancer. Therefore, this paper evaluates the efficacy and safety of Olaparib in the treatment of ovarian cancer by synthesizing the existing clinical trial system, in order to provide clinical evidence.

## Methods and Materials

We followed the Preferred Reporting Items for Systematic Reviews and Meta-Analyses (PRISMA) guidelines to report our meta-analysis. We systematically searched domestic and foreign literatures on the treatment of recurrent platinum sensitive ovarian cancer and systematically evaluated the efficacy and safety of Olaparib in the treatment of ovarian cancer.

### Search Strategy

We use a variety of retrieval tools to conduct a comprehensive literature search. (1) Computer literature database search: ①Chinese search terms included “Olaparib”, “ovarian cancer”, “clinical trials” and “randomized controlled studies”, etc. ②English keywords included “PARP inhibitors”, “ovarian cancer”, “clinical trials” and “randomized controlled studies”, etc. ③Different combinations of PubMed, Cochrane Library, Embase and EBSCO evidence-based medicine databases were searched, including title, abstract and keywords, and the search period was from self-establishment to January 2022.

### Study Selection

In order to further systematically evaluate the efficacy and safety of Olaparib in the treatment of ovarian cancer, the following inclusion criteria were used: (1) Included population: the diagnosis and inclusion criteria were consistent with the diagnostic criteria of recurrent platinum sensitive ovarian cancer, regardless of gender and race; (2) Literature type: prospective or retrospective studies; (3) Interventions: Experimental group: olaparib alone and combined with conventional chemotherapy; Control group: conventional chemotherapy or placebo. The administration and treatment of other basic diseases were not considered. (4) Outcome measures: ① Overall survival (OS); ② Adverse reactions at all levels (including nausea, fatigue, vomiting, diarrhea, abdominal pain, headache); ③ Adverse reactions above grade III (including nausea, fatigue, vomiting, diarrhea, abdominal pain and headache). Meanwhile, the following exclusion criteria were used: (1) “Non RCT or clinical trial”, “short case report”, “review”, “animal experiment” and other types of papers; (2) Repeatability study; (3) Non-Chinese and English literature; (4) Reports without data; (5) Unable to obtain full-text literature.

### Data Extraction and Quality Assessment

Data were extracted from the eligible studies included according to the PRISMA statement: author’s name, year of publication, type of publication (such as publication poster and abstract), country patient sample size, HRs and 95%CI of olaparib treatment window, OS was defined as spanning from randomization to death, adverse reactions of any grade and above grade 3. The Newcastle-Ottawa scale (NOS) was used to evaluate the quality of the literature (5), and the quality of the included studies was evaluated according to the following 8 criteria: (1) the representativeness of the exposure cohort; (2) the non-exposure cohort Selection; (3) Determination of exposure method; (4) No subject had an outcome event before the start of the study; (5) Comparability of exposure cohort and non-exposure cohort; (6) Evaluation of outcome events; (7) Whether the follow-up time is long enough; (8) Whether the follow-up is complete. Documents rated 7-9 points are considered “high” quality, 4-6 points are “fair”, and 3 points or lower are considered “low”. The quality evaluation is carried out independently by two researchers and cross-checked. If there is a disagreement, the third researcher is requested to assist in the resolution.

### Statistical Analysis

Meta-analysis was performed using RevMan 5.2 software provided by the Cochrane Collaboration. All the HRs included in the study were pooled together to provide an overall effect size. Cochrane χ^2^ test was used to analyze the heterogeneity between studies, and I^2^ was used to evaluate the heterogeneity. When P > 0.1 and I^2^ < 50%, there was no statistical heterogeneity for RCTs, and the fixed-effect model was used. On the contrary, the random effect model was adopted on the premise of excluding clinical heterogeneity. An inverted funnel plot was used to analyze publication bias, and sensitivity analysis was conducted for each included literature. The experimental bias of included literature was also discussed.

## Results

### Search Results and Patient Characteristics

**Figure 1:**
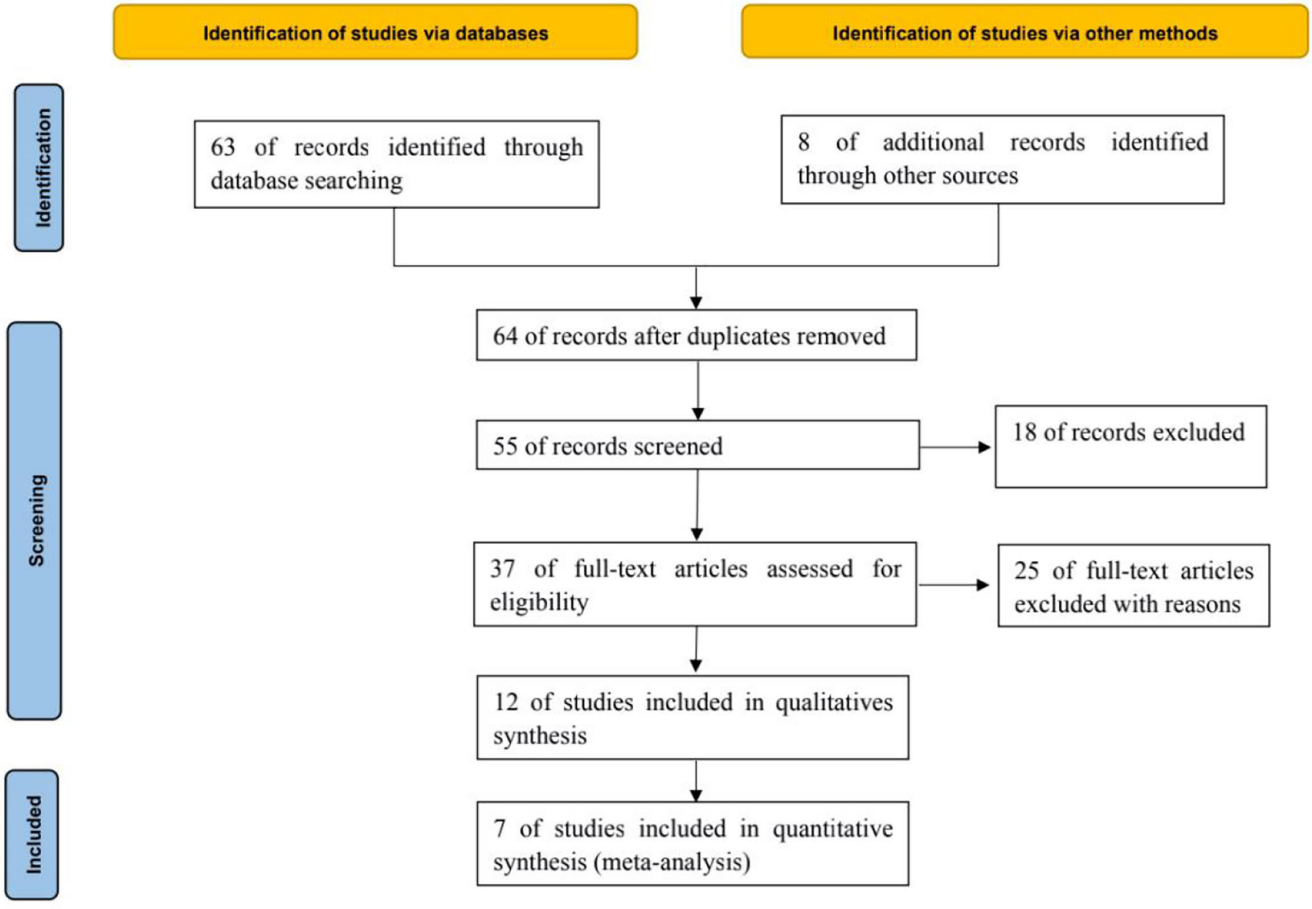
PRISMA Flow chart of article selection.

A total of 63 relevant literatures were obtained through database retrieval, and 8 were obtained through other methods, all of which were in English. 16 duplicate literatures were excluded, 9 inconsistent literatures were excluded from reading titles and abstracts, and 25 literatures were excluded from reading full text. After layer by layer screening, 7 RCTs (6–12) were finally included, including 2406 patients, including 1497 in the treatment group and 909 in the control group, as shown in **Figure 1**.

The basic data of 7 literatures (6–12) are relatively complete, and the baseline data such as patient age, gender and treatment strategy are balanced. The difference between the two groups is not statistically significant and comparable, as shown in **Table 1**.

**Table 1:** Basic characteristics of included studies (6–12).

First Author	Year	Phase	ID of study	Group	Number of cases	Intervention	mOS,Olaparib+ vs Olaparib-(months)	HR for OS[95% CI]	p-Value for OS	Quality
Richard T. Penson (6)	2020	III	NCT00628251	EG	151	olaparib 300mg po 2/d	NA	NA	NA	7
				CG	72	chemotherapy				
Jonathan Ledermann (7)	2012	II	NCT00753545	EG	136	olaparib 400mg po 2/d	29.7 vs 29.9	0.94[0.63,1.39]	0.75	8
				CG	129	placebo				
K. Moore (8)	2018	III	NCT01844986	EG	260	olaparib 300mg po 2/d	51.8 vs 15.1	0.95[0.60,1.53]	NA	7
				CG	130	chemotherapy				
Ray-Coquard (9)	2019	III	NCT02477644	EG	537	olaparib 300mg po 2/d	NA	NA	NA	7
				CG	269	chemotherapy				
Amit M Oza (10)	2014	II	NCT01081951	EG	81	olaparib 200mg po 2/d	33.8 vs 37.6	1.17[0.79,1.73]	0.44	8
				CG	81	chemotherapy				
Jonathan A Ledermann (11)	2016	II	NCT00753545	EG	136	olaparib 400mg po 2/d	29.8 vs 27.8	0.73[0.55,1.69]	0.025	7
				CG	129	placebo				
Andrés Poveda (12)	2021	III	NCT01874353	EG CG	196 99	olaparib 300mg po 2/d placebo	51.7 vs 38.8	0.74 [0.54,1.00]	0.054	9

EG, Experimental group; CG, Control group; NA, Not available; po, oral; OS, Overall survival; HR, Hazard ratio

### Meta-Analysis Results

#### Effect of Olaparib on OS in Patients With Ovarian Cancer

**Figure 2:**
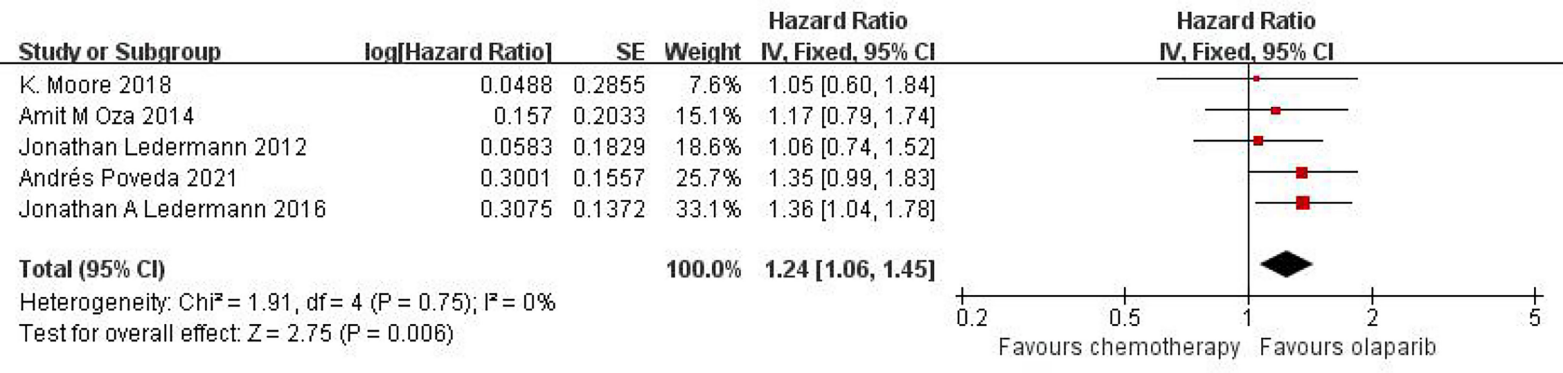
Meta-analysis results of OS between control group and olaparib group.

Five studies can obtain OS data for heterogeneity analysis. I^2^ = 0%, P = 0.75. There is no statistical heterogeneity among studies. Fixed effect model is used for analysis. The results showed that HR = 1.24 (95%CI = 1.06-1.45, P = 0.006), suggesting that Olaparib can significantly prolong OS in patients with ovarian cancer, as shown in **Figure 2**.

#### Any Grade of Adverse Reactions Caused by Olaparib

**Figure 3:**
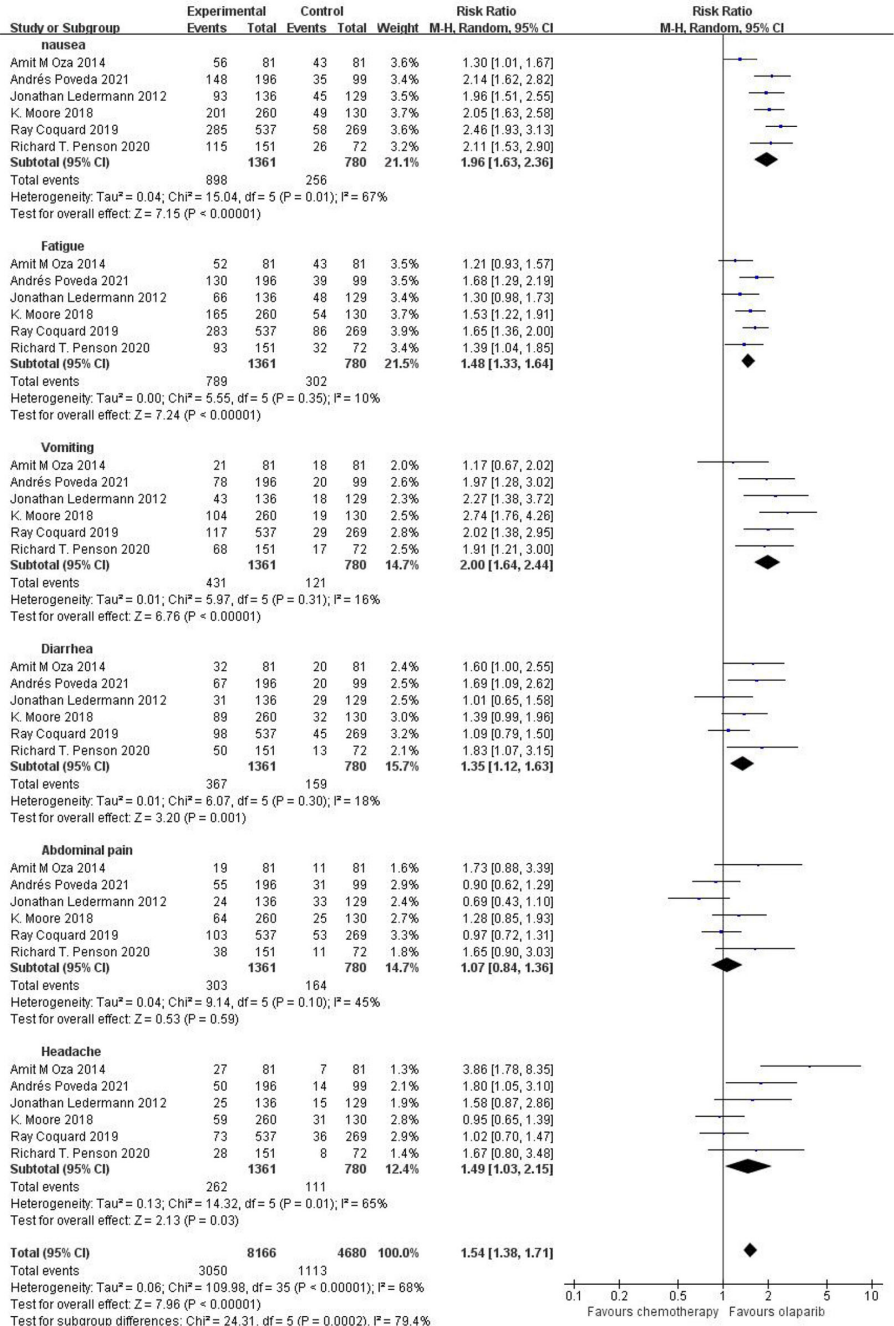
Meta-analysis results of adverse reactions of any grade between control group and olaparib group.

Six studies compared any grade of adverse reactions, including nausea, fatigue, vomiting, diarrhea, abdominal pain, and headache, and meta-analysis of the results showed that the relative risk of any grade of adverse reactions was higher in the olaparib group than in the control group [RR=1.54, 95%CI (1.38, 1.71), P=0.0002], and had statistical significance (P < 0.05). Subgroup analysis showed that, compared with the control group, only abdominal pain [RR=1.07, 95%CI (0.84, 1.36), P=0.03] and headache [RR=1.49, 95%CI (1.03, 2.15), P=0.03] in olaparib group had no significant difference, and nausea [RR=1.96, 95%CI (1.63, 2.36), P<0.00001], fatigue [RR=1.48, 95%CI (1.33, 1.64), P<0.00001], vomiting [RR=2.00, 95%CI (1.64, 2.44), P<0.00001] and diarrhea [RR=1.35, 95%CI (1.12, 1.63), P=0.001] were statistically significant (P < 0.05), as shown in **Figure 3**. This suggests that the use of olaparib is more prone to the above-mentioned adverse reaction symptoms.

#### Grade 3 or Higher Adverse Reactions Caused by Olaparib

**Figure.4:**
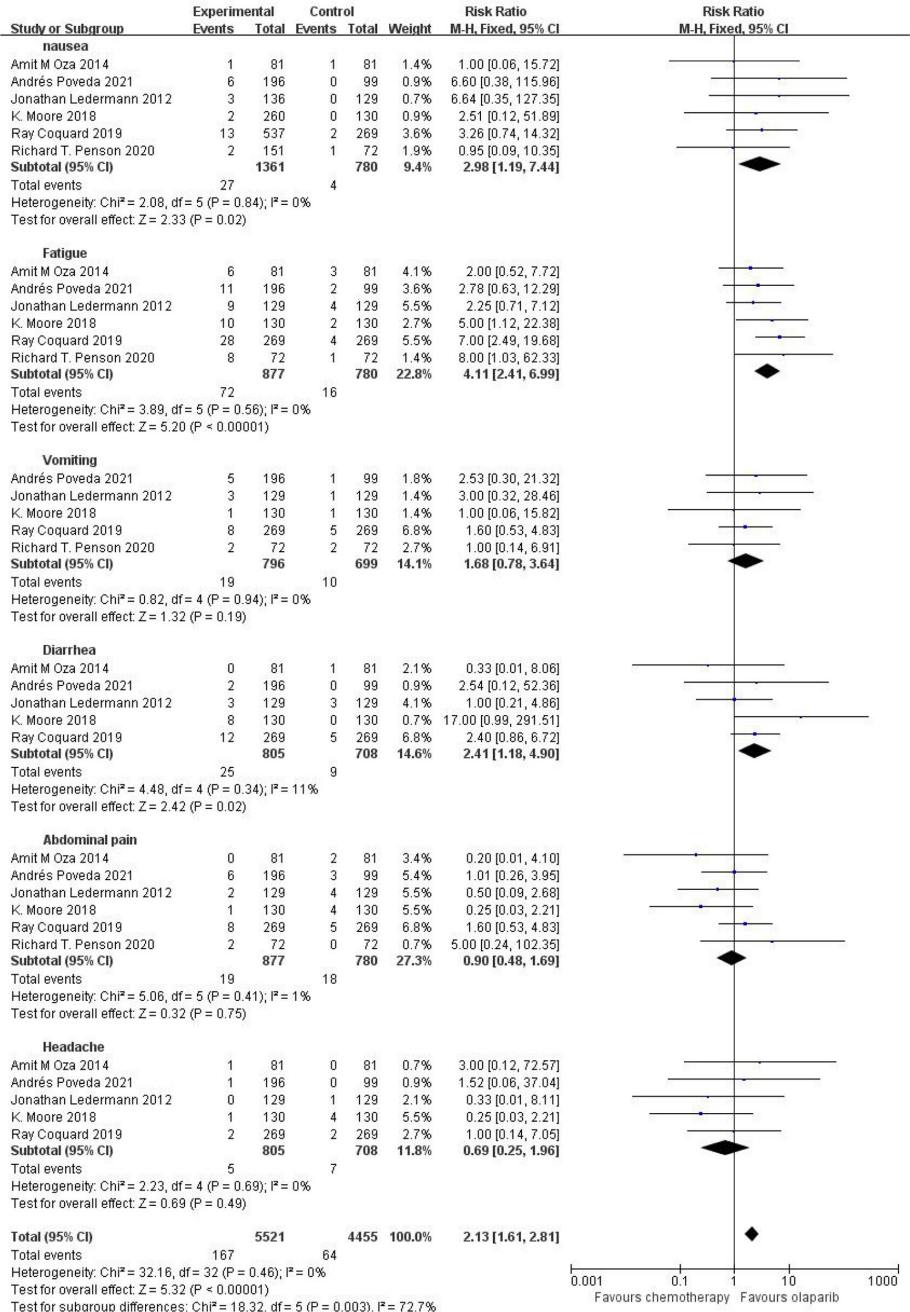
Meta-analysis results of adverse reactions above grade 3 between control group and olaparib group.

Six studies compared grade 3 or higher adverse events, including nausea, fatigue, vomiting, diarrhea, abdominal pain and headache, in the treatment group with the control group. The results showed that the relative risk of adverse reactions of any grade in the olaparib group was higher than that in the control group [RR=2.13, 95%CI(1.61, 2.81), P=0.003], with statistical significance (P<0.05). Subgroup analysis showed that, compared with the control group, olaparib group only vomiting [RR=1.68, 95%CI (0.78, 3.64), P=0.19], abdominal pain [RR=0.90, 95%CI (0.48, 1.69), P=0.75] and headache [RR=0.60, 95%CI (0.25, 1.96), P=0.49], nausea [RR=2.98, 95%CI (1.19, 7.44), P=0.02], fatigue [RR=4.11, 95%CI (2.41, 6.99), P < 0.00001] and diarrhea [RR=2.41, 95%CI (1.18, 4.90), P=0.02] (P < 0.05), as shown in **Figure 4**, suggesting that olaparib is more likely to cause severe grade III or above adverse reactions, mainly including fatigue and nausea and diarrhea symptoms.

### Publication Bias

**Figure 5:**
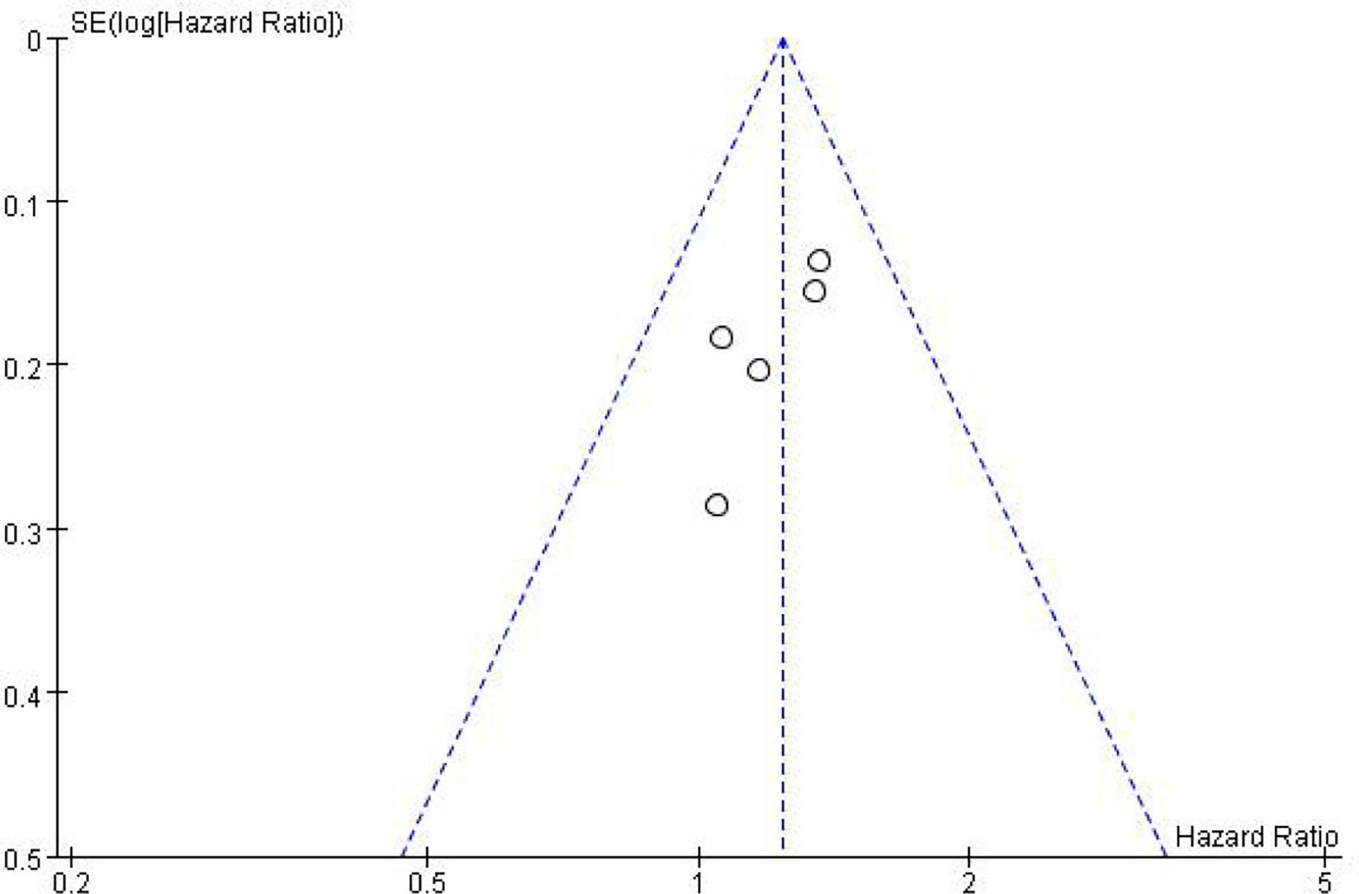
Inverted funnel plot of OS.

In addition to meta-analysis and comparison of overall survival and toxicity indices between the two groups, an inverted funnel plot was drawn for the included studies, which was symmetrical, suggesting a small or unbiased presence (in **Figure 5**).

### Sensitivity Analysis

In the sensitivity analysis, the combined HRs of OS did not change significantly after each exclusion of one study, and there was no significant difference in the analysis results before and after the exclusion, suggesting that all meta-analysis results were stable.

## Discussion

Among gynecological malignancies, the death rate of ovarian cancer is higher. In 2020, there were 55342 new cases of female ovarian cancer in China, with 37519 deaths, accounting for 67.8% (13). Clinically, platinum and paclitaxel are the first choice chemotherapy drugs for ovarian cancer patients, and they are sensitive to the initial chemotherapy regimen. However, ovarian cancer patients have a high probability of recurrence and death within 3 years. The loss of sensitivity of cancer cells to the cytotoxicity of antitumor drugs seriously restricts the efficacy of antitumor drugs, which is also the main reason for the failure of ovarian cancer treatment. In recent years, robotics has been widely used in cancer treatment, especially in minimally invasive surgery, which plays a good role in the prognosis of cancer patients. Many studies have highlighted its safety and further advantages in performing some of the most complex surgical procedures. Valerio Gallotta et al. (14, 15) confirmed that robotic aortic lymphadenectomy plays a good role in gynecological cancer, especially in ovarian cancer patients, robotic surgery can improve the prognosis of patients. Breast cancer susceptibility genes (BRCA) is an important DNA homologous repair gene, which plays a major role in the normal cell DNA repair mechanism. However, BRCA mutation can lead to homologous recombination defects and cause tumor recurrence. BRCA has also become one of the main factors for risk assessment and prognosis of ovarian cancer in recent years (16). The BRCA gene has been shown to be a good predictor of ovarian cancer prognosis. Claudia Marchetti et al. (17) and Daniela Rivera et al. (18) showed that the IMPLEMENTATION of NGS based BRCA tumor tissue detection in FFPE ovarian cancer specimens confirmed that BRCA gene could predict the prognosis of patients. Genetic testing can be developed for patients through collaboration between pathology and genetics.

Olaparib is a potent oral poly (ADP-ribose) polymerase inhibitor that also acts against BRCA1 or BRCA2 mutations. In numerous clinical trials, olaparib therapy has shown significant antitumor activity in ovarian cancer patients with and without BRCA mutations. In platinum-sensitive recurrent serous ovarian cancer, maintenance treatment with olaparib significantly improved progression-free survival compared with the control group (hazard ratio [HR] 0.35 [95%CI 0.25-0.49]; p<0.0001), the greatest clinical benefit was recorded in patients with BRCA mutations (HR 0.18 [95%CI 0.10-0.31]; p<0.0001) (13). Preclinical data show that Olaparib may enhance the efficacy of DNA damage chemotherapy, including platinum containing drugs such as carboplatin. Carboplatin combined with paclitaxel is widely used in the treatment of platinum sensitive, recurrent and high-grade serous ovarian cancer. Many studies have shown that olaparib combined with chemotherapy has a significant effect in the treatment of advanced ovarian, breast and other solid tumors.

The results showed that the adverse reactions of olaparib group at all levels and above were higher than those of the control group. These adverse reactions mainly included nausea, fatigue, vomiting, diarrhea, abdominal pain and headache. Our subgroup analysis showed that except for abdominal pain, headache and vomiting, other adverse reactions were statistically significant. The above suggests that although olaparib can prolong the overall survival of patients in the clinical treatment of ovarian cancer, the adverse reactions similar to the above should not be ignored, especially the clinical symptoms such as nausea, fatigue and vomiting. If they are aggravated, they should be treated symptomatically in time, which will help to improve the discomfort and compliance of patients during medication.

In conclusion, the analysis of 6 clinical trials shows that olaparib is an ideal drug regimen for the treatment of recurrent platinum sensitive ovarian cancer, and its overall survival time is longer than that of the control group, but its incidence of adverse reactions is high, which should be paid attention to in clinical use. This study still needs to carry out more large sample and multi center RCTs to further provide basis for clinical medication.

## Data Availability Statement

The original contributions presented in the study are included in the article/supplementary material. Further inquiries can be directed to the corresponding author.

## Author Contributions

We declare that all authors made fundamental contributions to the manuscript. All authors contributed to the study conception and design. Database search and data analysis was conducted by QC and XL. Study selection and data extraction were performed by XL and ZZ. The manuscript was written by QC. TW reviewed the manuscript. All authors read and approved the final manuscript.

## Conflict of Interest

The authors declare that the research was conducted in the absence of any commercial or financial relationships that could be construed as a potential conflict of interest.

## Publisher’s Note

All claims expressed in this article are solely those of the authors and do not necessarily represent those of their affiliated organizations, or those of the publisher, the editors and the reviewers. Any product that may be evaluated in this article, or claim that may be made by its manufacturer, is not guaranteed or endorsed by the publisher.
